# Artificial intelligence-assisted clinical exome sequencing: Insights and outcomes from 822 pediatric diagnoses

**DOI:** 10.1016/j.gimo.2026.104488

**Published:** 2026-07-31

**Authors:** Yinghong Pan, Patrick Danley, Tamar Kramer, Mehrdad Noruzinia, Karen Buser, Alexander N. Yatsenko, Daniel Bellissimo, Fen Guo

**Affiliations:** 1 UPMC Clinical Genomics Laboratory, Magee-Womens Hospital, Pittsburgh, PA; 2 Department of OB/GYN, School of Medicine, University of Pittsburgh, Pittsburgh, PA; 3 Department of Genetics, Yale University School of Medicine, New Haven, CT; 4 Department of Pathology, School of Medicine, University of Pittsburgh, Pittsburgh, PA; 5 Department of Genetics, School of Public Health, University of Pittsburgh, Pittsburgh, PA; 6 High Throughput Genomics Core for Clinical and Translational Medicine, University of Pittsburgh, Pittsburgh, PA

**Keywords:** Artificial intelligence, Clinical exome sequencing, Diagnostic yield, HPO

## Abstract

**Purpose:**

This retrospective study examined the clinical and genetic characteristics of pediatric patients undergoing clinical exome sequencing (ES) and evaluated the performance of a commercially available artificial intelligence (AI) platform that was integrated into our analysis pipeline.

**Methods:**

ES was performed in 822 consecutive patients at a single clinical laboratory. AI-based tools were used to jointly assess genetic information and the proband’s Human Phenotype Ontology terms to support variant prioritization during the initial case review.

**Results:**

A definitive molecular diagnosis was established in 22% (181 of 822) of index cases, while 40% (325 of 822) had variants of uncertain significance. Among those with a definitive diagnosis, 93% (168 of 181) had a single finding and 7% (13 of 181) had multiple findings. Of the 152 reported pathogenic/likely pathogenic variants in the fully resolved cases, 98.7% were successfully flagged by AI, and 75.0% ranked among the top 10 “most likely” variants.

**Conclusion:**

Clinical ES provides a substantial diagnostic yield in complex pediatric disorders. Integration of AI-powered platforms can accelerate phenotype-driven variant prioritization and facilitate rare disease diagnostics, but underscores the need for careful validation and optimization in clinical workflows.

## Introduction

Clinical exome sequencing (ES) is a commonly used molecular diagnostic test for patients with rare genetic disorders or cases with no definitive diagnosis by traditional genetic technologies. The American College of Medical Genetics and Genomics (ACMG) guidelines strongly recommend ES or genome sequencing (GS) as a first- or second-tier test for pediatric patients with unexplained intellectual disability, developmental delay, or congenital anomalies.^1^ The diagnostic yield of ES ranges from 20% to 40%, varying by clinical indication and cohort selection.^2^, ^3^, ^4^, ^5^, ^6^ ES performed as a trio, analyzing the proband along with both parents, enables the detection of de novo and compound heterozygous variants and helps to deprioritize rare variants inherited from an unaffected parent.^2^ ES also allows unbiased identification of multiple pathogenic/likely pathogenic (P/LP) variants that contribute to the patient’s phenotype or uncovers more than 1 genetic condition in a single patient, even when multiple disorders are not clinically apparent.

The analysis of ES/GS data is conventionally performed by trained clinical geneticists using a series of variant filtering and interpretation steps supported by bioinformatics tools. Despite its clinical value, this process remains labor-intensive, time-consuming, and subject to variability based on individual expertise and judgment.^2^^,^^3^ An average of 20 to 40 hours of expert time is often required to evaluate a clinical ES case.^7^ With the rising demand for genomic testing and the need for expedited turnaround times, variant prioritization and interpretation have become a critical bottleneck in the diagnostic workflow. One solution is using artificial intelligence (AI) tools that are rapidly evolving for application in ES/GS analysis.^8^, ^9^, ^10^, ^11^, ^12^ However, their clinical utility in real-world cases remains largely understudied.

Since 2021, our laboratory has incorporated a commercial AI platform into our clinical ES workflow to support variant prioritization and interpretation. Here, we present results from 822 pediatric patients who underwent ES between January 2021 and July 2024 at our clinical laboratory, where they were reviewed and the AI performance in variant prioritization was evaluated.

## Materials and Methods

### Patient information

Between 2021 and 2024, patient samples were referred to our laboratory by physicians and genetic counselors for suspected genetic diseases. Informed consent was obtained for each proband through the test requisition form, which also included an option for receiving secondary findings, as recommended by ACMG guidelines.^13^^,^^14^

### Clinical indication and Human Phenotype Ontology terms

At the time of testing, genetic counselors from our laboratory conducted a review of the clinical indication for testing, provided detailed phenotypic data and clinical records of the patients, and then entered a list of Human Phenotype Ontology (HPO) terms into the analysis platform for each proband undergoing ES. For this study, a genetic counselor retrospectively reviewed the clinical indications and assigned each proband a primary clinical indication category, including central nervous system (including seizures, autism, brain malformations, and intellectual disability/developmental delay [ID/DD]), peripheral nervous system, hearing, eye (vision/structural), cardiovascular, respiratory, abdomen, genitourinary, skeletal, muscle, connective tissue, skin, endocrine, growth, multiple congenital anomalies (MCA), immune, neoplasm, metabolism, mitochondrial, and bleeding disorders (Supplemental Figure 1).

### Exome sequencing and variant calling

Genomic DNA was extracted from peripheral blood for the proband and from peripheral blood or saliva samples for family members, following standard DNA extraction and library preparation protocols in our laboratory. DNA libraries were enriched for all coding regions and splice sites (±10 bp) in the exome using the Sophia DDM Whole Exome Solution (Sophia Genetics), a custom oligonucleotide-based capture method, followed by sequencing using 2×150 bp paired-end reads on Illumina next-generation sequencing (NGS) systems (Illumina). Reads were mapped to the human genome reference GRCh38. Sequence variants and copy number variants (CNVs) were detected using Sophia Genetics DDM software. Variants were called at a minimum coverage of 10× and an allele fraction of 20% or higher. The 10% quantile of target region coverage was 125 to 200×; more than 99.5% of the targets were covered at >25×. Mitochondrial reads were mapped to the mitochondrial DNA reference NC_012920.1, with 100% of the targets covered at >1000×. Mitochondrial variants were called at allele fractions of 5% or higher.

### Variant prioritization, analysis, and reporting

Variants were automatically annotated and prioritized using the Emedgene online AI platform (Illumina). Filtered variants were manually reviewed, interpreted, and classified by a genomic analyst using the ACMG/Association for Molecular Pathology guidelines.^13^ Selected variants were further reviewed and correlated by a genetic counselor and a laboratory director prior to finalizing them into clinical reports.

In this supervised analysis workflow, AI was implemented as a tertiary analysis platform to support phenotype-driven variant prioritization, clinical review, variant classification, and reporting. During the initial case review, AI was used to jointly assess the sequencing variants from the proband and available family members alongside the proband’s clinical symptoms, which were represented by HPO terms. In addition to the “most likely” and “candidate” bins, user-defined preset bins (Supplemental Material) were implemented in the workflow to filter any additional variants/genes that fit the patient’s clinical presentation. All flagged variants were manually reviewed.

Reported P/LP variants and variants that did not meet the laboratory quality criteria were confirmed by Sanger sequencing. Some single-nucleotide variants (SNVs) were excluded from confirmation after meeting the laboratory threshold for quality criteria and confirmation policy. CNVs were confirmed by long-range polymerase chain reaction (PCR), droplet digital PCR, breakpoint PCR, microarray, multiplex ligation-dependent probe amplification, or another orthogonal method, whenever possible. Sequence variants and CNVs classified as likely benign or benign were not confirmed or reported. In addition to primary diagnostic findings, ACMG secondary findings were captured in a user-defined preset bin and were reported if patients had consented to receiving them.^14^

### Statistical analysis

For group comparisons (age groups, trio/duo/solo groups, and different HPO number groups), overall χ^2^ tests were performed to assess their global differences in diagnostic yield. When the overall test was not significant, pairwise comparisons were conducted using Fisher exact test. To account for multiple pairwise testing, Bonferroni correction was applied within each comparison set. The adjusted statistical significance was set at *P* < .0083 for age groups and *P* < .0167 for trio/duo/solo groups (2-tailed).

## Results

### ES diagnostic yield and impact factors

In this retrospective study, ES results from 822 pediatric patients (0 to 18 years old) between 2021 and July 2024 at our laboratory were reviewed along with their clinical features to evaluate the clinical utility of ES.

**Figure 1:**
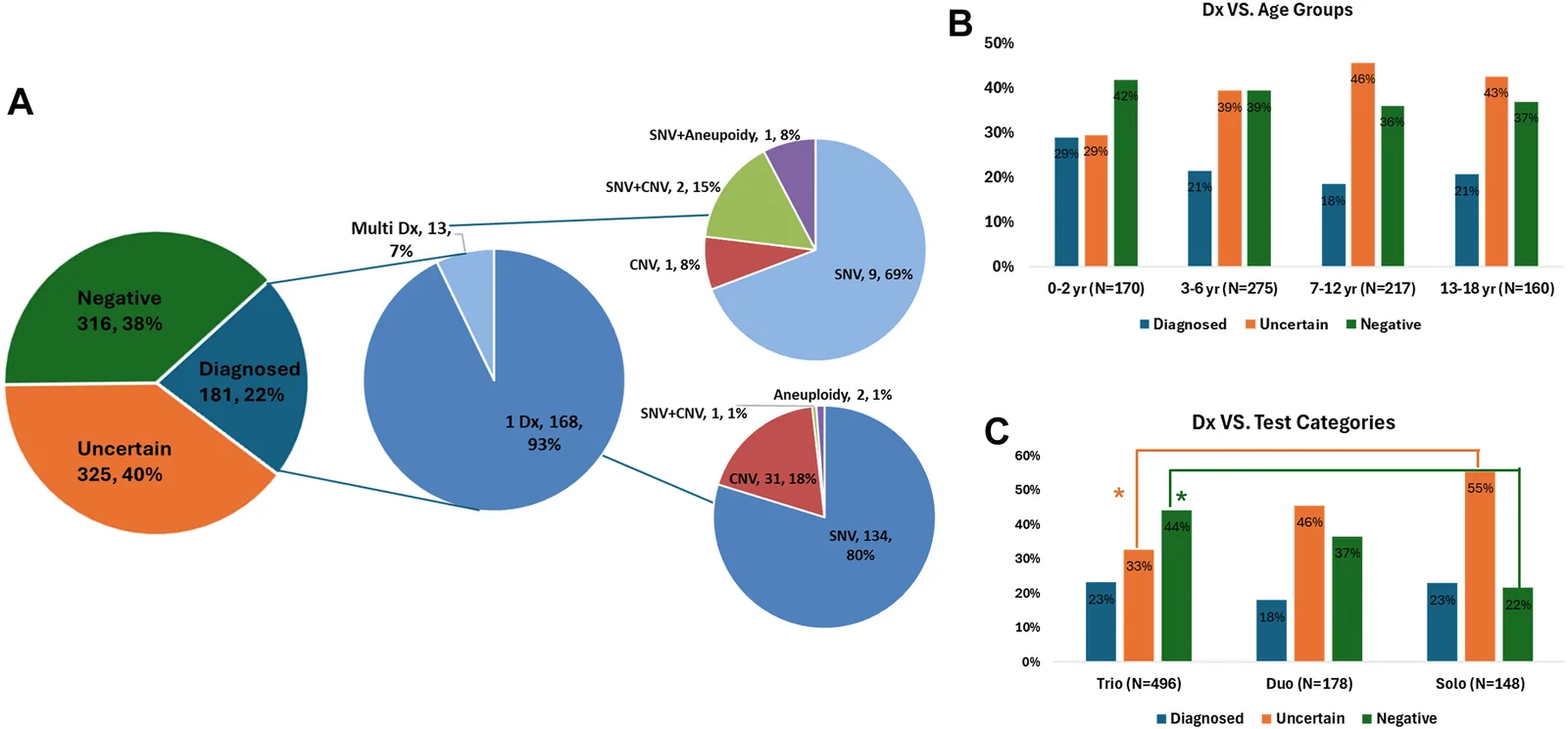
**Diagnostic yield and impact factors**. A. The overall percentage of cases receiving definitive diagnosis, cases with VUS findings, and negative cases. The pathogenic variant types and the percentages in the diagnosed cases. B. Diagnostic yield vs age group. C. Diagnostic yield vs test category. ∗ adjusted *P* value < .0167. VUS, variant of uncertain significance.

Overall, 22% (181 of 822) of index cases received a definitive molecular diagnosis consistent with their primary indications for testing (diagnosed cases). An additional 40% of cases (325 of 822) had uncertain results, with only variants of uncertain significance (VUS) findings of dominant inherited genes or 1 P/LP and 1 VUS finding of recessive genes associated with the clinical presentation (uncertain cases), and 2% of cases (16 of 822) had a gene variant from the ACMG-defined secondary finding list.^14^ Among patients with a definitive molecular diagnosis, 93% (168 of 181) had a single finding and 7% (13 of 181) had multiple potentially relevant genetic findings (Table 1). SNVs/indels were the most common type of genetic variant and accounted for >80% of all the diagnostic cases, while CNVs were identified in nearly 20% of the cases (Figure 1A). Several genes were identified as recurring diagnoses in 5 or more cases (Supplemental Table 1).

**Table 1:** Multiple diagnostic findings: Cases had 2 or more positive diagnostic outcomes

Case ID	Age	Primary Clinical Indication	HPO_Terms	Test	Molecular Diagnosis and Inheritance
ES_0004	0	immune	Failure to thrive, Leukodystrophy, Infantile spasms, Seizure, Immunodeficiency, Hypertonia, Eczema, Abnormality of skin pigmentation, Ichthyosis, Skin rash, Feeding difficulties, Lymphadenopathy, Hypsarrhythmia	trio	*UBA5* (AR)-P and P; *FLG* (AD/AR)-P and P
ES_0131	7	CNS-autism	Autistic behavior, Global developmental delay, Atrial septal defect, Patent ductus arteriosus, Hypotonia, Hydronephrosis, Constipation, Failure to thrive, Abnormal chromosome morphology, Choroid plexus papilloma, Optic nerve hypoplasia, Renal duplication, Preauricular skin tag, Brain imaging abnormality, EEG abnormality, Hydroureter, Recurrent otitis media, Kyphosis, Simple febrile seizure, Intraventricular hemorrhage, Premature atrial contractions, Low-set ears, Overfolded helix, Short palpebral fissure, Epicanthus, Long eyelashes, Highly arched eyebrow, Synophrys, Widely spaced teeth, Drooling, Hypertrichosis, Prominent fingertip pads, Abnormal vertebral morphology	duo	*EHMT1* (AD)-LP; *CACNA1A* (AD)-LP; chr16 dup-P
ES_0364	13	CNS-DD	Abnormal facial shape, Premature ventricular contraction, Underfolded helix, Single transverse palmar crease, Myopia, Epistaxis, Genu valgum, Developmental regression, Asthma, Micrognathia, Anxiety, Bridged palmar crease, Constipation, Prolonged neonatal jaundice, Inguinal hernia, Clinodactyly, Simple ear, Microcephaly, Long eyelashes, Short stature, Failure to thrive, Limited wrist movement, Bulbous nose, Cutis marmorata, Syndactyly, Cryptorchidism, Pes planus, Intrauterine growth retardation, Premature birth, Hyperactivity, Narrow forehead, Carious teeth, Bruising susceptibility, Frontal upsweep of hair, Infra-orbital crease, Abnormally large globe, Limited elbow extension	solo	*PTPN11* (AD)-P; *TRIO* (AD)-LP
ES_0485	14	MCA	Hyperactivity, Intellectual disability, Blue sclerae, Low-set ears, Abnormality of connective tissue, Hypertrichosis, Tall stature, Syringomyelia, Dental crowding, Joint hypermobility, Tapered finger, Craniosynostosis, Global developmental delay, Synophrys, Hypermelanotic macule, Sacral dimple, Posteriorly rotated ears, Pectus carinatum, Thick eyebrow, Widely spaced teeth, Autistic behavior, Arachnodactyly, Strabismus, Cleft palate, Genu recurvatum, Accelerated skeletal maturation, Kyphosis, Short palpebral fissure, Cleft lip, Abnormal facial shape	trio	chr2 del-LP; chr16 dup-LP
ES_0563	9	CNS-seizure	Macrocephaly, U-Shaped upper lip vermilion, Esotropia, Myopia, Falls, Seizure, Absent speech, Feeding difficulties, Hypotonia, Dystonia, Global developmental delay, Constipation, Gastroesophageal reflux, Nystagmus, Pointed chin, Myopathic facies, Strabismus, Encephalopathy, Failure to thrive, Bruising susceptibility, Temperature instability, Cerebral white matter agenesis	trio	*KCNQ2* (AD)-P; *ALPL* (AR)-LP and VUS
ES_0607	5	CNS-autism	Autistic behavior, Absent speech, Developmental regression, Global developmental delay, Abnormality of brain morphology, Premature birth, Nonimmune hydrops fetalis, Prolonged neonatal jaundice, Patent ductus arteriosus, Patent foramen ovale, Abnormal facial shape, Epistaxis, Optic neuropathy, Strabismus, Nystagmus, Hypotonia, Abnormal diffusion weighted cerebral MRI morphology, Supraventricular tachycardia, Thin upper lip vermilion, Widely spaced teeth, Downslanted palpebral fissures, Wide nasal bridge, Prominent nasal tip, Simple ear, Premature thelarche, Dry skin, Abnormal corpus callosum morphology, family hx cancer, hx respiratory distress	solo	*OCA2* (AR)-P and LP; *FLG* (AD/AR)-P and P
ES_0683	16	CNS-DD/ID	Astigmatism, Cataract, Nystagmus, Myopia, Sensorineural hearing impairment, Seizure, Muscle weakness, EEG abnormality, Migraine, Hemiplegia, Pes planus, Intellectual disability, Global developmental delay, Arrhythmia, Decreased glucose-6-phosphate dehydrogenase level in blood	duo	*G6PD* (XL)-P and VUS; *ATP1A3* (AD)-LP
ES_0731	12	Skin	Macrocephaly, Photophobia, Telangiectasia, Albinism, Prolonged QT interval, Vertigo, Nevus, Fatigue, Staring gaze, Hypertropia, Family history of long QT syndrome, Oculocutaneous albinism, Hypoplasia, Disorder of optic nerve, Anomalous head position, Strabismic amblyopia, Pseudoptosis, Red mole, Dark mole, Abnormal facial shape, Congenital nystagmus, Recurrent infections, recurrent otitis media	trio	*TYR* (AR)-P and P; *KCNQ1* (AD)-P
ES_0757	4	GU	Hypospadias, Astigmatism, Hyperpigmentation of the skin, Spasticity, Global developmental delay, Hypertonia, Abnormal facial shape, Prolonged neonatal jaundice, Tip-toe gait, Cryptorchidism, Recurrent otitis media, Small scrotum, Intrauterine growth retardation, Ankle clonus, Testicular dysgenesis, cafe au lait, hyperactivity, Epicanthus, Long palpebral fissure, Infra-orbital fold, Broad nasal tip	solo	*SRSF1* (AD)-LP; chr16 dup-LP
ES_0839	2	CNS-brain malformations	Intrauterine growth retardation, Prolonged neonatal jaundice, Failure to thrive, Short stature, Aggressive behavior, Autistic behavior, Atypical behavior, Delayed speech and language development, Developmental regression, Global developmental delay, Ataxia, Patent ductus arteriosus, Ventricular septal defect, Constipation, Feeding difficulties, Gastroesophageal reflux, Microcephaly, Blue sclerae, Cafe-au-lait spot, Dermatographic urticaria, Staring gaze, Abnormal diffusion weighted cerebral MRI morphology, Allergy, Acne, Urticaria, Headache, Bruising susceptibility, Abnormal facial shape, Trisomy X syndrome, Thin corpus callosum, Slow weight gain, Dilated R ventricle, Nonspecific signal change on brain MRI, Chronic idiopathic urticaria	duo	*IGF1R* (AD)-LP; Trisomy X-P
ES_0852	2	MCA	Premature birth, Global developmental delay, Delayed speech and language development, Hypotonia, Conductive hearing impairment, Macrocephaly, Recurrent infections, Microtia, bilateral urinary tract dilation, Ureteropelvic junction obstruction, Anisocoria, Single umbilical artery, flexibility, Pes planus, Atresia of the external auditory canal	duo	*FBN1* (AD)-LP; *SMARCC1* (AD)-LP
ES_0873	1	CNS-ID/DD	Short stature, Global developmental delay, Hypermelanotic macule, Hypotonia, Syndactyly, Umbilical hernia, Orofacial cleft, Exotropia, G6PD deficiency, In utero drug exposure, Plagiocephaly, Abnormal facial shape	solo	*SETD5* (AD)-LP; *G6PD* (XL)-P and VUS
ES_0874	5	CNS-autism	Prolonged neonatal jaundice, Failure to thrive, Autistic behavior, Global developmental delay, Eczema, Hypotonia, Microcephaly, Hx paraspinous hemangioma, fatty infiltration of filum terminale, Hypermetropia, Astigmatism, Gait disturbance, Aspiration, pseudoesotropia, firm stools, Cafe-au-lait spot, bruising from falls, Acne, Frequent falls, Abnormal facial shape, Unsteady gait	solo	*SPG7* (AD)-P; *SRRM2* (AD)-LP

*AD,* autosomal dominant; *AR,* autosomal recessive; *CNS,* central nervous system; *EEG*, electroencephalography; *GU,* genitourinary; *HPO,* human phenotype ontology; *ID/DD,* intellectual disability/developmental delay; *LP,* likely pathogenic; *P,* pathogenic; *MCA,* multiple congenital anomalies; *MRI*, magnetic resonance imaging; *VUS*, variants of uncertain significance; *XL,* X-linked.

Among different age groups, the highest diagnostic yield (29%, 49 of 170) was observed in the 0 to 2 years age group, but without statistical significance (χ^2^ test, *P* = .144) (Figure 1B). Among all cases, 60% (496 of 822) were trio cases, 22% (178 of 822) were duos, and 18% (148 of 822) were solos. The diagnostic yield was similar across trio (23%), duo (18%), and solo (23%) analyses (χ^2^ test, *P* = .445). Trios exhibited greater diagnostic certainty, with fewer uncertain results (33% vs 55%, Fisher exact test, *P* < .0001) and a higher negative result rate (44% vs 22%, Fisher exact test, *P* < .0001) compared with solos. Duo cases (uncertain, 46%; negative, 37%) did not differ significantly from either trios or solos (Figure 1C).

### Phenotypic spectrum and HPO-guided diagnostic yield

The most frequent phenotype categories indicated in this cohort included autism, ID/DD, MCA, connective tissue abnormalities, and seizures (Supplemental Figure 1).

**Figure 2:**
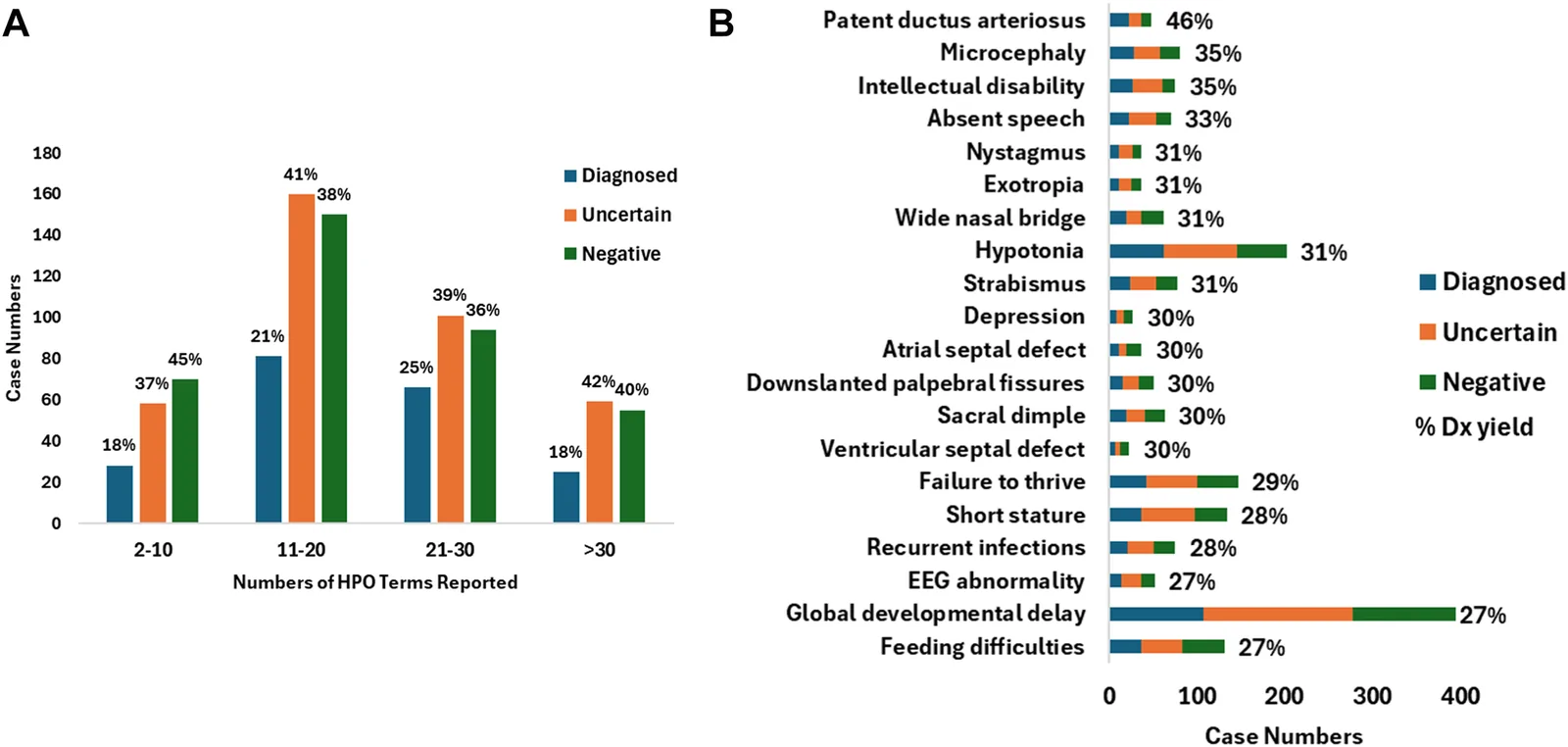
**HPO terms reported**. A. Distribution of numbers of HPO terms reported and diagnostic yield in each group. B. Top 20 HPO terms associated with the highest diagnostic yield. HPO, Human Phenotype Ontology.

The patients’ specific phenotypes were typically annotated with HPO terms. In this cohort, the majority of cases were reported with 11 to 20 (41%) or 21 to 30 (28%) HPO terms on the test requisition forms, while 16% of the cases reported 2 to 10 HPO terms and 15% reported over 30 HPO terms. The diagnostic yields did not differ significantly among groups with varying numbers of reported HPO terms (χ^2^ test *P* = .3134). Although slightly lower diagnostic yields were observed in cases with fewer than 10 or more than 30 terms (18%) compared with other groups (21 to 25%), this difference was not statistically significant (Fisher exact test *P* = .1218) (Figure 2A).

Among the most frequently reported HPO terms, “Abnormal facial shape” and “Global developmental delay” each occurred in approximately 400 cases. Both were associated with diagnostic yields over 25% when less specific and combined with other terms (Supplemental Figure 2). Certain more specific HPO terms were particularly enriched among positive findings: “Patent ductus arteriosus” (46%, 22 of 48), “Microcephaly” (35%, 22 of 48), “Intellectual disability” (35%, 26 of 75), “Absent speech” (33%, 23 of 70), “Strabismus” (31%, 24 of 77), “Hypotonia” (31%, 62 of 202), “Wide nasal bridge” (31%, 19 of 62), “Exotropia” (31%, 11 of 36), “Nystagmus” (31%, 11 of 36), and “Ventricular septal defect” (30%, 7 of 23) (Figure 2B).

### AI performance for assisting analysis

**Figure 3:**
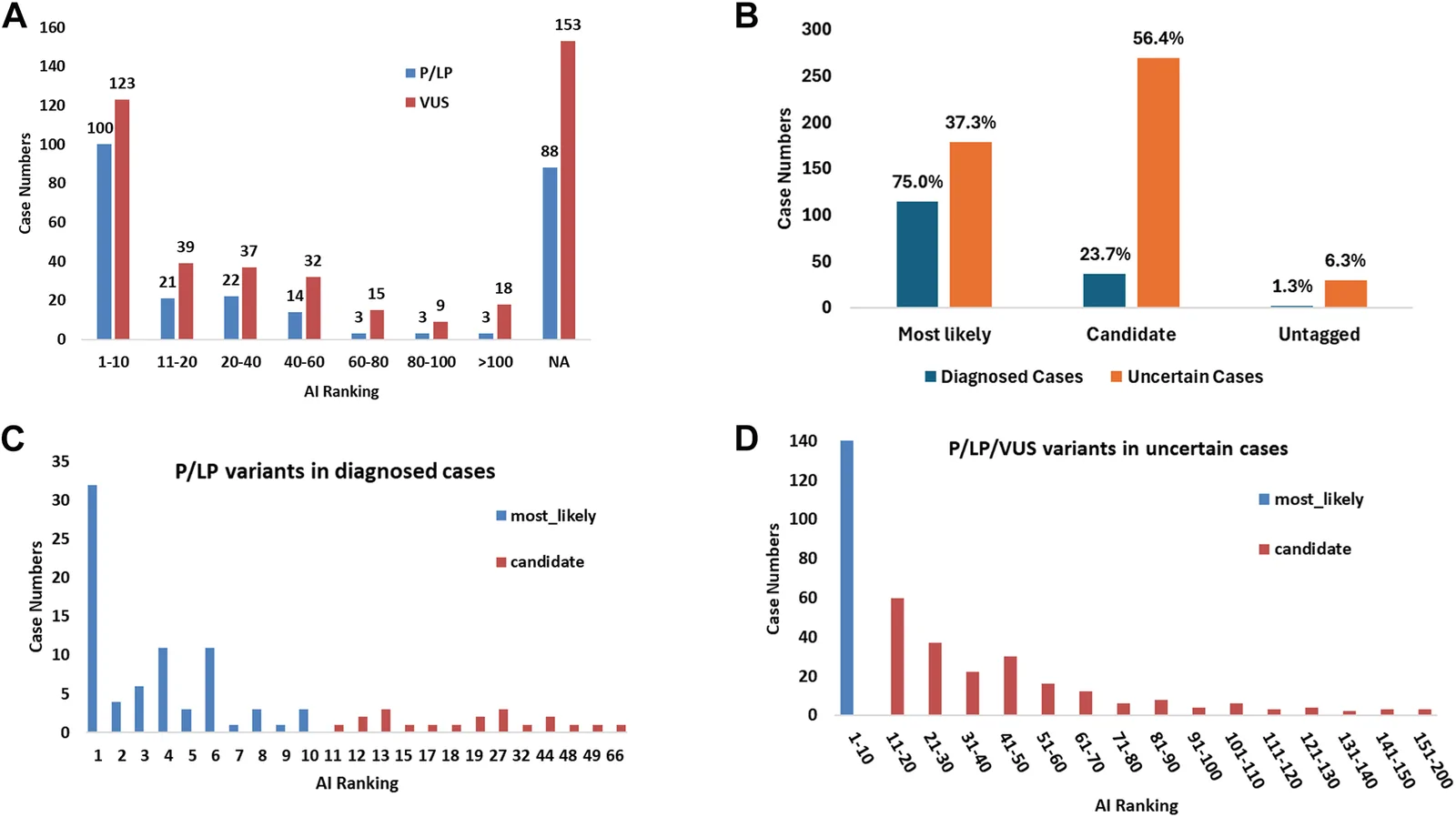
**AI performance in reportable SNVs**. A. AI ranking distribution of P/LP and VUS variants in all cases. B. AI tags in diagnosed and uncertain cases. C. AI rankings of P/LP variants in diagnosed cases. D. AI rankings of all variants in uncertain cases. AI, artificial intelligence; P/LP, pathogenic/likely pathogenic; SNV, single-nucleotide variant; VUS, variant of uncertain significance.

We systematically examined the AI performance on the 680 reportable SNVs, with 254 P/LP variants and 426 VUS. For 241 variants (88 P/LP and 153 VUS) analyzed using an older version of the platform, the AI classified them only as “most likely” or “candidate” without assigning a numerical rank. For variants analyzed with the updated version, the AI both categorized and numerically ranked them: variants tagged ‘most likely’ were ranked 1 to 10, whereas “candidate” variants were ranked ≥11 (up to approximately 200) (Figure 3A).

Among the 152 reported P/LP variants in the diagnosed cases, 75.0% (114 to 152) were tagged as “most likely,” 23.7% (36 to 152) were tagged as “candidate,” and 1.3% (2 to 152) were untagged due to a low variant allele fraction caused by a highly homologous region or pseudogene presence (Figure 3B). While among the 477 reported SNVs in the uncertain cases, 37.3% (178 of 477) were tagged as “most likely,” 56.4% (269 of 477) were tagged as “candidate,” and 6.3% (30 to 477) were untagged but selected for reporting via preset customized filters (Figure 3B).

For the diagnosed cases with a numerical AI ranking, the median ranking was 3 for the 75 ”most likely” P/LP variants, with 42.7% (32 of 75) ranking 1; and the median AI ranking was 19 for the 20 “candidate” P/LP variants, with a maximum ranking of 66 (Figure 3C). For the uncertain cases with AI ranking, the median ranking was 4 for the 134 “most likely” SNVs and 38 for the 176 “candidate” SNVs (Figure 3D).

### ES value in cases with prior test results

**Figure 4:**
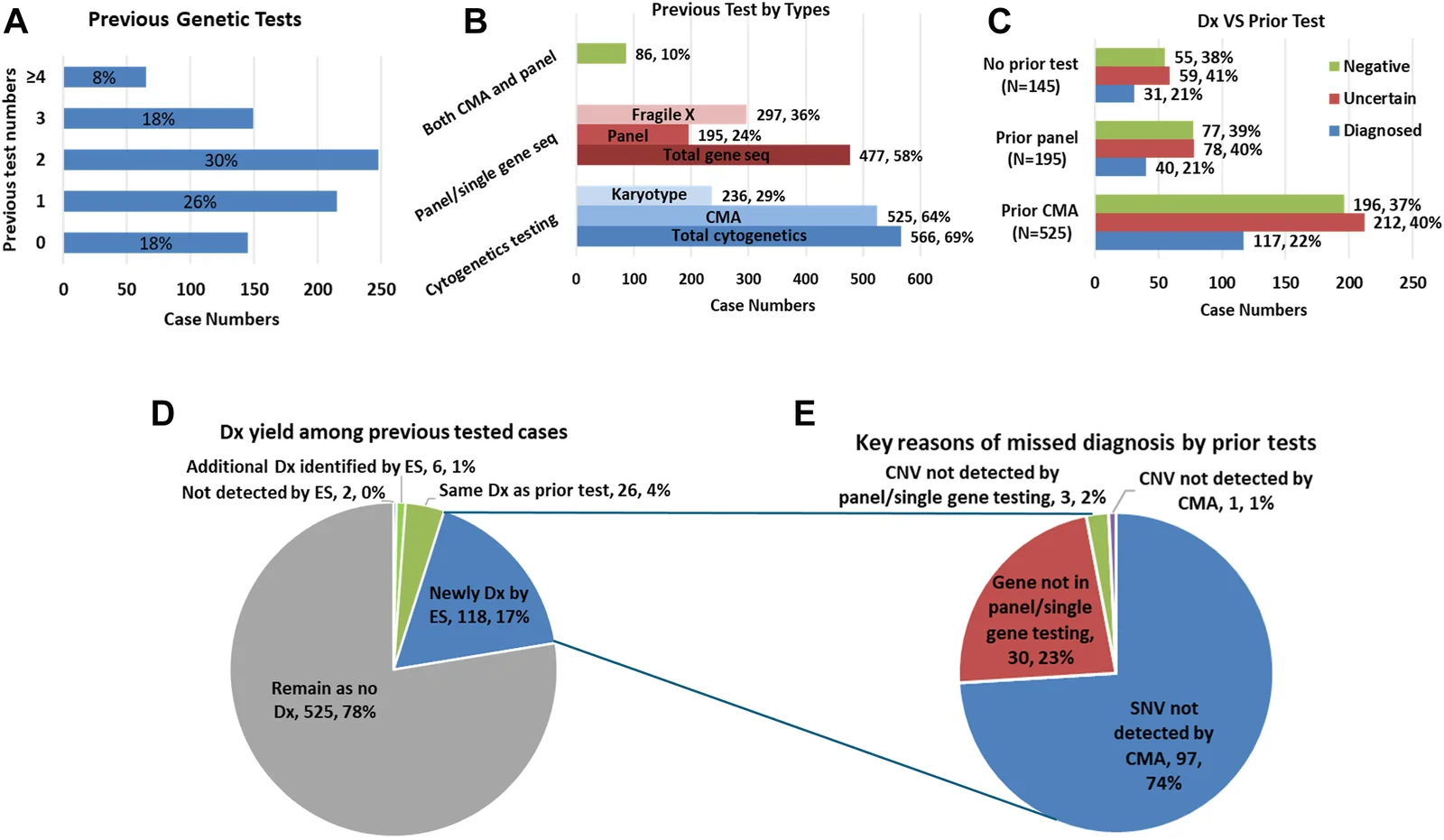
**Diagnostic yield of ES as a first-tier test verse as a follow-up test**. A. ES cases with previous genetics tests by test numbers. B. ES cases with previous genetic tests by major test category. C. Diagnostic yield among ESwith no prior test, with prior panel test and with prior CMA. D. ES diagnosis among previous tested cases (*n* = 677). E. Key reasons contribute to the missed diagnosis by prior ES (*n* = 118). CMA, chromosomal microarray; ES, exome sequencing.

Among the 822 index pediatric cases, 82% of cases had 1 or more previous genetic tests performed and ES was ordered as a follow-up test, while only 18% used ES as the first-tier test (Figure 4A). The most frequent prior test was chromosomal microarray analysis (CMA), performed in 64% (525 of 822) of cases. Other previous cytogenetic tests included karyotype (29%, 236 of 822) and fluorescence *in situ* hybridization (2.4%, 20 of 822). In addition, 58% (477 of 822) of cases had previous gene panel or single-gene tests, including 24% (195 of 822) with a panel test, 36% (297 of 822) with Fragile X testing, and at least 20 cases with other single-gene tests. Other prior tests included methylation studies, multiplex ligation-dependent probe amplification, and diepoxybutane analysis. Notably, 10% (86 of 822) of cases had both CMA and panel testing (Figure 4B and C).

Thirty-four cases with prior genetic tests had already received a positive diagnostic finding that partially explained the phenotype, and ES was requested to explore additional genetic etiology. ES successfully confirmed these previous diagnostic findings in 94% (32 of 34) of cases. The 2 unconfirmed cases were imprinting disorders with methylation pattern changes. Importantly, ES further identified additional genetic etiology in 6 of the 34 patients, resulting in multiple diagnoses (Figure 4D, Table 1).

Among 118 cases that tested positive and were newly diagnosed by ES, diagnoses were missed by prior tests due to the following reasons: SNVs were not detected by CMA (97 cases); the relevant genes were not included in the panel or single-gene test (30 cases); small CNVs were not detected by the panel or single-gene test (3 cases); a small CNV was missed by CMA (1 case) (Figure 4E).

### Representative illustrative diagnostic cases (variants described in the results and discussions are summarized in Table 2)

#### Multiple diagnosis case

Case ES_0131 was a 6-year-old male with a history of poor weight gain, global developmental delay, autism, behavioral problems, stridor, optic nerve hypoplasia, hypotonia, duplicated kidney, megaureter, segmentation anomaly, atrial septal defect, patent ductus arteriosus, umbilical hernia, and abnormal brain magnetic resonance imaging (MRI) and electroencephalography. He also had unique dysmorphic features, including bilaterally low-set ears with overfolded helices, a left preauricular tag, short palpebral fissures, bilateral epicanthal folds, long eyelashes, arched eyebrows, mild synophrys, widely spaced teeth, significant sialorrhea, mild hypertrichosis of the back, and prominent bilateral fetal finger pads. Family history was positive for intellectual disability, developmental delays, hyperactivity, cardiac and renal defects, hearing loss, cancers, and other diseases. A clinical microarray test done 4 years ago identified a 16p11.2 microduplication as a VUS, which was not detected in the maternal sample. The clinical features associated with 16p11.2 microduplication overlapped but did not perfectly match those of the proband. Therefore, a duo ES (proband + mother) was ordered. The 16p11.2 microduplication (chr16: 29609982–30307538, arr[GRCh38] 16p11.2(29609982_30307538)×3, approximately 698 kb) was confirmed by ES and was upgraded to pathogenic, as sufficient evidence for triplosensitivity has emerged over the years. In addition, ES identified a splicing variant c.2018+1G>T in the *EHMT1* gene (NM_024757.5), which is associated with autosomal dominant Kleefstra syndrome 1 (OMIM 610253), and explained his distinctive facial features, cardiac defects, and renal defects. ES also identified a maternally inherited nonsense variant c.5803C>T (p.Gln1935Ter) in the *CACNA1A* gene (NM_001127222.2), which is associated with autosomal dominant developmental and epileptic encephalopathy 42 (OMIM 617106). AI tagged the *EHMT1* and *CACNA1A* variants as “most likely.” The 16p11.2 CNV was also successfully selected by AI in a user-defined preset bin that specifically filters for CNVs associated with phenotypically matched diseases. This case illustrates the strength of ES in providing a comprehensive molecular diagnosis in patients with multiple disease etiologies.

#### Role of parental studies in variant reclassification

Case ES_0683 was a 16-year-old male with a history of global developmental delay, intellectual disability, alternating hemiplegia, migraines, seizures, left wedge cataract, myopia, astigmatism, nystagmus, unilateral hearing loss, irregular heartbeat, abnormal MRI and electroencephalography, G6PD deficiency, muscle weakness, and dysmorphic features. His family history was positive for intellectual disability, psychiatric disorders, cancers, sickle cell, and G6PD. Methylation PCR, karyotype, microarray, and metabolic laboratory test results were all normal, and single-gene tests for *CACNA1A, ATP1A2,* and *SCN1A* also returned negative results. Duo ES (proband + mother) initially identified 2 maternally inherited *G6PD* variants, c.202G>A (p.Val68Met) and c.376A>G (p.Asn126Asp) (NM_001042351.3), as well as VUS in *MAPK8IP3* c.2366C>T (p.Thr789Met) (NM_001040439.2), and *ATP1A3* c.2516T>C (p.Leu839Pro) (NM_152296.5); neither of the latter was found in the maternal sample. *MAPK8IP3* is associated with neurodevelopmental disorder with or without variable brain abnormalities (OMIM 618443). *ATP1A3* is associated with cerebellar ataxia, areflexia, pes cavus, optic atrophy, and sensorineural hearing loss (CAPOS, OMIM 601338) and alternating hemiplegia of childhood 2 (OMIM 614820). Both genes are associated with neurological diseases that phenotypically match the proband. Later, the paternal sample was received for segregation study. The *MAPK8IP3* variant was found to be paternally inherited and downgraded to likely benign. The *ATP1A3* variant was not found in the paternal sample, indicating it was de novo. The variant was upgraded to likely pathogenic. The *MAPK8IP3* and *ATP1A3* variants were both tagged as ”most likely” by AI. The *G6PD* variants were selected in a user-defined preset bin for variants with known pathogenicity. This case highlights the importance of parental studies in VUS classification, especially for ES/GS.

#### Small CNV detected by ES but missed by CMA

Case ES_0351 was a 2-year-old boy with a history of global developmental delay, hypotonia, sialorrhea, failure to thrive, alternating intermittent exotropia, retinal vascular abnormality, eczema, eosinophilic esophagitis, atopic dermatitis, benign enlargement of extra-axial subarachnoid spaces on brain MRI, and retractile testes. The previous CMA returned a normal result. A de novo 14 kb deletion (chr18:52928791–52942797; arr[GRCh38] 18q21.1(52928791_52942797)x1) encompassing exon 11 of 20 of the *TCF4* gene (NM_001083962.2) was identified by ES and confirmed with droplet digital PCR. Pathogenic variants in the *TCF4* gene are associated with autosomal dominant Pitt-Hopkins syndrome (OMIM 610954), which was consistent with the clinical presentation of the proband. This CNV in *TCF4*, which had not been previously reported in CMA and was absent from the gnomAD population database, was expected to result in a frameshift leading to a truncated protein or loss of expression due to nonsense-mediated decay. This case provides a strong example demonstrating that ES can have higher sensitivity than CMA for detecting small CNVs, particularly when probe density and distribution across the affected genomic region are limited in CMA.

## Discussion

ES has been demonstrated to increase diagnostic yield, especially in the pediatric population, improving treatment options and providing risk information for families.^2^^,^^15^^,^^16^ In this study, we present the results from 822 pediatric patients who underwent ES between January 2021 and July 2024 at our clinical laboratory. The 3.5-year accrual period reflects the pace of case accumulation in a modest-volume clinical laboratory and the need for careful validation of technical updates. During this period, workflow refinements, selective automation, and bioinformatics enhancements were implemented to improve throughput and efficiency while maintaining clinical standards. The overall diagnostic yield in this cohort was 22%, and an additional 40% had at least 1 VUS finding reported. This study did not include a direct comparison to a pre-AI workflow, limiting conclusions regarding incremental diagnostic yield. Historical comparisons were confounded by changes in case mix and analytic methods over time. The observed 22% yield fell within the reported ranges for heterogeneous clinical exome cohorts.

### Factors impacting the diagnostic yield

Factors associated with diagnostic yield included patient age, family members segregation, testing approach, and clinical phenotype.

Younger age was generally linked to an earlier onset and a more severe clinical presentation, both of which contribute to a higher likelihood of achieving a molecular diagnosis.^17^, ^18^, ^19^ The diagnostic yield was reported as 27% to 50% in some studies for critically ill neonatal cases.^20^^,^^21^ In our cohort, the diagnostic yield for infants (0-year-old, *n* = 36) was 22%, which was lower than the 29% diagnostic rate for children aged 1 to 2 years (*n* = 134) in this cohort. Under the current molecular test workup and infrastructure at our hospital, most of the symptomatic neonatal intensive care unit cases are sent out for STAT GS given its more uniform coverage, improved detection of structural and CNVs, and utility in time-sensitive clinical decision-making. Therefore, our cohort included limited infant cases with relatively milder symptoms, which may skew the molecular diagnostic yield in this group. As sequencing costs decrease, bioinformatics infrastructure matures, and, most importantly, insurance coverage for GS significantly improves, we anticipate a continued transition from exome to genome-based testing.

Trio testing has also been reported to lead to a higher certainty diagnostic rate compared with solo ES testing,^2^^,^^22^^,^^23^ while some studies have shown that they achieve a similar diagnostic yield with only modest improvements in trios without statistical significance.^24^^,^^25^ In our cohort, trio cases had a comparable diagnostic yield to solo cases. However, a greater diagnostic certainty (a higher negative rate and a lower uncertain result rate) was observed for the trio group compared with the solo group. The comparable diagnostic yield between trio and solo ES may reflect our clinically integrated workflow, in which we collaborated closely with clinicians on each case and offered segregation studies as a complementary follow-up after ES. For solo cases with uncertain variants that could benefit from segregation analysis, we routinely performed Sanger testing if parental samples were available (as illustrated by case ES_0683), thereby achieving a relatively high diagnostic yield in solo sequencing and reducing the potential incremental gain from trio testing. However, this approach increases both turnaround time and analytical workload. In contrast, trio ES allows the immediate prioritization of de novo or compound heterozygous variants in clinically relevant genes and rules out some VUS when inherited from healthy parents, thus eliminating the need for Sanger segregation studies, reducing the number of variants requiring curation, and offering a lower cost per diagnosis when considering analysis time alone.

Certain clinical indications or phenotype categories may have higher genetic attributes and lead to a higher diagnostic yield, such as 36.3% (45 of 124) for ID/DD and 25.2% (30 of 119) for MCA compared with 0% for Respiratory or Neoplasm (Supplemental Figure 1). The patient’s phenotypes, typically described using HPO terms, dictate the interpretation of ES genetic data and impact the diagnostic yield. However, the diagnostic yield did not correlate with the number of HPO terms; instead, it was associated with the specificities of the HPO terms provided. Certain specific HPO terms involving facial dysmorphic features (including “Microcephaly,” “Strabismus,” “Wide nasal bridge,” “Exotropia,” “Nystagmus,” “Sacral dimple,” and “Downslanted palpebral fissures”), congenital heart defects (including “Patent ductus arteriosus,” “Ventricular septal defect,” and “Atrial septal defect”), or neurodevelopmental abnormalities (including “Intellectual disability,” “Absent speech,” “Depression,” and “Global developmental delay”), have been found to be associated with a higher diagnostic yield (27% to 46%) in our cohort. This was in line with the ACMG guideline recommending ES as a first- or second-tier test for pediatric patients with congenital anomalies or ID/DD.^1^ In contrast, some nonspecific or ambiguous HPO terms were associated with a low diagnostic yield, such as “Abdominal pain” (7%), “Tremor”(12%), and “Fatigue” (15%). AI-based analysis tools usually heavily rely on HPO terms, using specificity-weighted fuzzy matching of HPO terms entered by users to identify the best match to diseases for variant prioritization during the initial case review.^26^^,^^27^ Although comprehensive phenotyping can improve gene matching, the inclusion of numerous or nonspecific terms may dilute the core phenotypic signal and affect variant ranking. While current algorithms partially mitigate this through hierarchical weighting, optimal performance relies on concise, clinically focused phenotyping. Custom HPO panels have been reported to increase the success rate and reduce the VUS for ES interpretation.^28^ Our result underscored the importance of phenotype-driven, well-defined specific HPO terms in AI-assisted ES analysis. In addition, ACMG secondary findings were assessed in parallel with indication-based analysis using a user-defined preset gene bin within the AI platform. Because this process was independent of HPO-driven prioritization, it provided a structured and efficient mechanism for evaluating medically actionable variants unrelated to the primary indication.

### ES as a comprehensive tool for molecular analysis

Although emerging evidence supports the clinical utility of ES for rare genetic diseases, in current practice, most insurance providers require preauthorization for an ES test, and over half of the ES tests ordered are denied coverage.^29^^,^^30^ In addition, insurance providers typically require evidence that simpler, less expensive tests, such as chromosomal microarray analysis, single-gene testing, or targeted gene panels, have been tried first to prove “medically necessary,” which makes the diagnostic odyssey “unnecessarily” long. The prior genetic tests for this cohort were also heavily affected by the established genetic test workup algorithm and infrastructure, especially the insurance reimbursement policies. In this study, most cases had previous genetic testing, and 26% of cases had ≥3 tests performed prior to ES. Most (94%) of the previous test results were confirmed by ES, suggesting that the earlier tests may have been avoidable. Moreover, ES successfully identified new molecular diagnoses for 14% (118 of 822) of the cohort. Among these cases, diagnoses were mostly missed before due to the limitations of those previous tests, such as genes or small CNVs not covered in the panel/single-gene test (eg, ES_0683), or CMA not being able to detect SNV or CNV due to the probe distribution. For example, ES confirmed the 16p11.2 microduplication detected by CMA and further detected 2 more pathogenic variants in *EHMT1* and *CACNA1A* in case ES_0131. One case with a small CNV detected by ES was missed by the previous CMA because the 14 kb deletion was too small and likely not sufficiently covered by microarray probes to be detected. Compared with CMA in CNV calling, ES has been reported to have higher sensitivity than low-resolution CMA and comparable sensitivity to high-resolution CMA.^31^ In addition, ES revealed multiple diagnoses in 13 cases. Overall, the technical superiority of ES led to an additional 18% (124 of 678) diagnostic rate on top of traditional cytogenetics or panel/single-gene testing. ES offers the advantage of comprehensive molecular analysis, which is essential to prevent laboratories from stopping too early before all phenotypically relevant variants are identified, ensuring that additional molecular diagnoses are not overlooked.^15^^,^^32^

Nevertheless, ES has its own limitations. In this study, ES could not confirm the results involving a case with Beckwith-Wiedemann syndrome and a case with Russell Silver syndrome (RSS). The Beckwith-Wiedemann syndrome case had loss of methylation at imprinting center 2 and normal methylation at imprinting center 1, as detected by a previous methylation study. The methylation status is beyond the regular ES detection capacity, which requires entirely different technologies that read epigenetic modifications.^33^ It is important to choose the most appropriate clinical test based on the clinical presentation and differential diagnosis. In the RSS case, instead of the uniparental disomy of chromosome 7 detected by previous CMA, ES identified compound heterozygous variants in the *UNC80* gene, which is associated with a disease that overlaps with the clinical presentation of RSS and may represent an additional etiology for the patient’s symptoms (Supplemental Material, ES_0787). Though ES has been reported to be able to accurately detect runs of homozygosity (ROH) and uniparental disomy,^34^ it requires a separate ROH caller, such as H(3)M(2) or BCFtools/RoH,^27^^,^^35^ which has not been incorporated into our analysis pipeline yet. Incorporating these ROH callers would allow for wider use of ES and for capturing potential multiple diagnoses in such cases.

### AI ranking efficiency and pitfall among ES analysis

With the advancement of NGS technologies and the lowering of costs, ES/GS tests have become more commonly used for comprehensive molecular diagnostic purposes. As the number of cases increases, analysis and interpretation become one of the time-limiting steps for high-throughput NGS. One of the major advantages of AI is its ability to reduce analysis time and effort while minimizing human bias and errors. In this study, we focused on diagnostic outcomes and systematically evaluated AI performance for SNVs in pediatric cases for ES analysis.

For the SNV analysis in diagnosed cases, the AI performance was particularly strong, with 98.7% of reported variants successfully flagged by AI as “most likely” or “candidate.” Moreover, with 42.7% of variants ranked by AI as 1 and a median rank of 3 in the “most likely” category, along with a median rank of 19 and a maximum rank of 66 in the “candidate” variants, our results demonstrated that the AI-based variant prioritization can substantially reduce the manual review burden and improve overall analysis efficiency. The only 2 untagged P/LP variants in diagnosed cases were both due to their low variant allele fractions (VAF), which were caused by the presence of highly homologous pseudogenes. The case ES_0470 (Supplemental Material) has a maternally inherited *CYP21A2* c.92C>T pathogenic variant with 11% VAF, which did not meet the variant quality control metrics and was initially filtered out. Similarly, another case with an *ABCC6* (NM_001171.6):c.196dup pathogenic variant (Table 2) with 15% VAF was filtered out. As both cases had highly specific phenotypes for gene-associated diseases, manually reviewing by removing all the filters and searching for the gene identified those variants, and long-range PCR followed by Sanger sequencing confirmed the findings. Homologous regions are known to pose technical hurdles in short-read NGS and can lead to false-positive and false-negative diagnostic errors in clinical tests if not handled correctly.^36^^,^^37^ ACMG and the College of American Pathologists require clinical laboratories to develop a strategy to distinguish between gene and pseudogene and document the accuracy of their testing.^38^^,^^39^ In the context of AI-assisted analysis, this issue may be addressed by creating an additional user-defined preset bin to screen genes with highly homologous regions and by lowering or removing the allele fraction filter.

In contrast, AI performance in cases with uncertain findings was less consistent: 6.3% (30 of 477) variants were untagged but selected for reporting via customized filter presets. This was primarily due to variable disease expressivity, incomplete penetrance, and limited supporting evidence for VUS. Given the inherent ambiguity of these variants, determining their clinical relevance remained biased and challenging. In addition to the AI-based approach, implementing additional presets that target known limitations may help reduce the risk of missed variants. However, this could slightly increase analysis time, so each laboratory should determine the best balance based on its workflow and priorities.

Other pitfalls we experienced in the AI-assisted analysis can be resolved by manual review of the AI-tagged variants. For example, in a case with an LP variant, c.819_822delinsGGTC (Table 2), in the *OCA2* gene (NM_000275.3), AI tagged 4 missense variants (c.819C>G, c.820T>G, c.821G>T, c.822G>C) as “candidates,” which required manual merging into the correct small indel variant according to Human Genome Variation Society nomenclature. This issue arises from the SNV caller processing variants separately, rather than from a limitation of the AI model. In principle, AI-based methods could readily recognize and automatically merge such indels. Another example was VUS variants in specific haplotypes. The VUS c.376A>G in the *G6PD* gene was a well-known variant that is observed in individuals with G6PD-deficient hemolytic anemia and was found at a high frequency in the general population (gnomAD v2.1.1: 6586 of 205,081 alleles or 3.2%). It was commonly held that this variant (referred to as A+) was not causative of disease unless it was found with one of several additional alleles (A-), including the c.202G>A variant.^40^^,^^41^ The c.376A>G variant was untagged by AI but was manually added by the analyst when the c.202G>A P variant was identified. This step can be further optimized by a small modification to the variant-calling algorithm, such as adding a preset bin for specific known haplotypes.

**Table 2:** Summary of variants described in results and discussion

Gene	HGNC ID	Transcript	Variant	Genomic Variants (g.)	Classification
*EHMT1*	24650	NM_024757.5	c.2018+1G>T	NC_000009.12:g.137776845G>T	LP
*CACNA1A*	1388	NM_001127222.2	c.5803C>T (p.Gln1935Ter)	NC_000019.10:g.13214537G>A	LP
*G6PD*	4057	NM_001042351.3	c.202G>A (p.Val68Met)	NC_000023.11:g.154536002C>T	P
*G6PD*	4057	NM_001042351.3	c.376A>G (p.Asn126Asp)	NC_000023.11:g.154535277T>C	VUS
*MAPK8IP3*	6884	NM_001040439.2	c.2366C>T (p.Thr789Met)	NC_000016.10:g.1765119C>T	VUS to LB
*ATP1A3*	801	NM_152296.5	c.2516T>C (p.Leu839Pro)	NC_000019.10:g.41970211A>G	VUS to LP
*CYP21A2*	2600	NM_000500.7	c.955C>T (p.Gln319Ter)	NC_000006.12:g.32040421C>T	P
*CYP21A2*	2600	NM_000500.7	c.92C>T (p.Pro31Leu)	NC_000006.12:g.32038514C>T	P
*ABCC6*	57	NM_001171.6	c.196dup (p.Ser66fs)	NC_000016.10:g.16221672dup	P
*OCA2*	8101	NM_000275.3	c.819_822delinsGGTC (p.Asn273_Trp274delinsLysVal)	NC_000015.10:g.28016172_28016175delinsGACC	LP
*UNC80*	26582	NM_032504.2	c.3203C>A (p.Ser1068Ter)	NC_000002.12:g.209839383C>A	LP
*UNC80*	26582	NM_032504.2	c.9355C>G (p.Pro3119Ala)	NC_000002.12:g.209994109C>G	VUS

*LB*, likely benign; *LP,* likely pathogenic; *P,* pathogenic; *VUS,* variant of uncertain significance.

CNVs were excluded from this AI evaluation because the CNV caller in use was developed by a different vendor (Sophia DDM), which was not fully integrated into our AI-assisted analysis pipeline (Emedgene), and the quality control metrics were not carried over. This emphasizes the importance of pipeline compatibility, where all quality metrics should be incorporated into a single system. Parallel analysis of SNVs and CNVs is considered best practice in clinical genomics.^42^, ^43^, ^44^ Automated CNV detection would further reduce manual review and enhance AI and ES use.

While prior studies have evaluated AI-assisted exome interpretation, our work examines its real-world, multiyear deployment within a clinical laboratory. This study evaluates AI-based variant ranking within a routine clinical review workflow using retrospectively analyzed cases. Our current workflow represents an AI-facilitated review model, in which AI-prioritized variants (“most likely” and “candidate”) are reviewed first, followed by a comprehensive manual review using user-defined preset bins. This staged approach preserves full analytical oversight while enabling systematic assessment of AI prioritization performance. Importantly, review of variants outside the AI-prioritized sets serves as an internal comparator, allowing evaluation of findings that would not be captured under an AI-only strategy and providing evidence-based support for defining review thresholds above which additional manual review may no longer be necessary.

Our results highlight the promise of AI-powered platforms in enhancing the efficiency of phenotype-driven variant prioritization and accelerating rare disease diagnostics, while also demonstrating the need for comprehensive validation and optimization, particularly in complex or ambiguous cases. These findings underscore the importance of understanding gene-disease relationships and highlight the value of detailed patient clinical information for clinical ES analysis and interpretation. Together, these observations emphasize that AI-based tools are most effective when integrated into a structured, expert-guided review process that leverages both computational prioritization and rich clinical context.

This study has limitations. As a single-center experience using a vendor-specific AI platform, generalizability may be limited because performance and efficiency can vary across institutions, workflows, and analytic infrastructures. Multicenter evaluations are important to assess broader applicability. In addition, future prospective studies that incorporate structured time-tracking and review thresholds informed by this analysis will be valuable for quantitatively assessing reductions in analysis time and overall workflow efficiency.

## Data Availability

The data supporting the conclusions of this article are included within the article and its additional files.

## Ethics Declaration

All probands were enrolled and sampled according to standard local practice in approved human participants protocols as part of routine clinical care at the University of Pittsburgh Medical Center hospital. Written informed consents for research use were obtained from the patients or their parents before the Exome Sequencing. This study was performed in accordance with the Declaration of Helsinki.

## Conflict of Interest

The authors declare no conflicts of interest.
