# Supplementary material for: Artificial intelligence-assisted clinical exome sequencing: Insights and outcomes from 822 pediatric diagnoses
Source: Genet Med Open. 2026 Jul 31;4:104488. doi: 10.1016/j.gimo.2026.104488 (PMC13545448; doi:10.1016/j.gimo.2026.104488)
Supplement: Supplementary file 1 [file mmc1.docx]

**Supplementary Materials**

**Additional Representative Cases**

**Case ES_0470** was a 5-month-old girl with a history of congenital adrenal hyperplasia, ambiguous genitalia, brachycephaly, and rhizomelia. Karyotype and FISH test results were normal. Trio ES revealed compound heterozygous in CYP21A2 gene (Supplementary Table 2), which is associated with congenital adrenal hyperplasia due to 21-hydroxylase deficiency (OMIM 201910). The ES result showed apparent variant allele fractions (VAF) of 25% and 11% for the paternally inherited variant c.955C>T p.Gln319Ter and maternally inherited variant c.92C>T p.Pro31Leu (NM_000500.7). Long range PCR followed by nested Sanger sequencing was able to confirm that the heterozygosity of each allele is around 50% originated from the *CYP21A2* gene instead of the pseudogene. AI was able to tag the *CYP21A2* c.955C>T p.Gln319Ter variant as ‘candidate’. And the c.92C>T p.Pro31Leu variant was filtered out in all the preset bins due to the 11% VAF lower than the cutoff value 20%.

***CYP21A2: c.955C>T p.Gln319Ter***

**A**

***CYP21A2: c.92C>T p.Pro31Leu***

**B**

***CYP21A2* VAF in case ES_0470. A)** IGV and Sanger confirmation for c.955C>T. **A)** IGV and Sanger confirmation for c.955C>T.

**Case ES_0787** was a 1-year-old girl with a history of IUGR, short stature, failure to thrive, relative macrocephaly, hypotonia, speech delay, blue sclera, sacral cleft, clinodactyly, hypoglycemia, dysmorphic features had UPD7 detected by previous CMA. ES found compound heterozygous variants (a *de novo* LP c.3203C>A p.Ser1068Ter and a maternally inherited VUS c.9355C>G p.Pro3119Ala, phase unknown; Supplementary Table 2) in *UNC80* gene (NM_032504.2), which is associated with autosomal recessively inherited infantile hypotonia with psychomotor retardation and characteristic facies-2 (IHPRF2, OMIM: 616801). This ES result for this case was reported as uncertain.

**User-Defined Preset Bins**

*Known Pathogenic*

*De Novo Trio*

*Homozygotes in AR*

*Compound Heterozygote*

*X-linked*

*Dominant Inheritance (Trio)*

*Heterozygote in AD*

*Mito-Known Pathogenic*

*Mito-MAF 0.5*

*Phenotype Match*

*CNV Phenomatch_High*

*CNV Phenomatch_All (<20kb)*

*CNV Phenomatch_All (>20kb)*

*CNV Dosage Sensitive Genes (>1kb)*

*CNV Dosage Sensitive Genes (<1kb)*

*CNV >5Mb*

*ACMG81-PATH*

*ACMG81-LOF*

**Supplementary Figures**

**Supplementary Figure 1. ES cases by Phenotype Categories.**

**Supplementary Figure 2. The top 20 frequently reported HPO and the Dx yield in those cases.**

**Supplementary Table**

| **Supplementary Table 1. Genes Reported Over 5 Times in the Cohort** | | | | | |
| --- | --- | --- | --- | --- | --- |
| **Gene** | **HGNC ID** | **Variant reported times** | **Associated clinical syndrome** | **OMIM** | **Inheritance** |
| *FLG* | 3748 | 36 | Ichthyosis vulgaris | 146700 | AD/AR |
| *G6PD* | 4057 | 11 | Hemolytic anemia, G6PD deficient (favism) | 300908 | XL |
| *TNRC6B* | 29190 | 10 | Developmental delay and Intellectual disability (TNRC6B) | 619243 | AD |
| *TET3* | 28313 | 10 | Beck-Fahrner syndrome | 618798 | AD/AR |
| *TNXB* | 11976 | 9 | Vesicoureteral reflux 8 | 615963 | AD/AR |
| *TRRAP* | 12347 | 9 | Developmental delay with or without dysmorphic facies and autism | 618454 | AD |
| *TRIO* | 12303 | 7 | Intellectual developmental disorder, autosomal dominant 44, with microcephaly | 617061 | AD |
| *PHIP* | 15673 | 6 | Chung-Jansen syndrome | 617991 | AD |
| *FBN1* | 3603 | 5 | Marfan lipodystrophy syndrome | 616914 | AD |
| *ARID1A* | 11110 | 5 | Coffin-Siris syndrome 2 | 614607 | AD |
| *CHD3* | 1918 | 5 | Snijders Blok-Campeau syndrome | 618205 | AD |
| *SPTBN1* | 11275 | 5 | Developmental delay, impaired speech, and behavioral abnormalities | 619475 | AD |
| *TANC2* | 30212 | 5 | Intellectual developmental disorder with autistic features and language delay, with or without seizures | 618906 | AD |
| *HUWE1* | 30892 | 5 | Intellectual developmental disorder, X-linked, Turner type | 309590 | XL |
| *VWF* | 12726 | 5 | von Willebrand disease, types 2A, 2B, 2M, and 2N | 613554 | AD/AR |

AD: autosomal dominant; AR: autosomal recessive; XL: X-linked.
